# Network of Spaces and Interaction-Related Behaviors in Adult Intensive Care Units

**DOI:** 10.3390/bs4040487

**Published:** 2014-12-01

**Authors:** Mahbub Rashid, Diane K. Boyle, Michael Crosser

**Affiliations:** 1 School of Architecture, Design and Planning, University of Kansas, 1465 Jayhawk Boulevard, Lawrence, KS 66045, USA; 2 Fay W. Whitney School of Nursing, University of Wyoming, Dept. 3065, 1000 E. University Ave, Laramie, WY 82071-3065, USA; E-Mail: dboyle@uwyo.edu; 3 School of Medicine, University of Kansas Medical Center, 3901 Rainbow Blvd., Mailstop 1022, Kansas City, KS 66160, USA; E-Mail: mcrosser@kumc.edu

**Keywords:** intensive care unit (ICU), interaction-related behaviors, space syntax, network of spaces, environmental visibility and accessibility

## Abstract

Using three spatial network measures of “space syntax”, this correlational study describes four interaction-related behaviors among three groups of users in relation to visibility and accessibility of spaces in four adult intensive care units (ICUs) of different size, geometry, and specialty. Systematic field observations of interaction-related behaviors show significant differences in spatial distribution of interaction-related behaviors in the ICUs. Despite differences in unit characteristics and interaction-related behaviors, the study finds that when nurses and physicians “interact while sitting” they prefer spaces that help maintain a high level of environmental awareness; that when nurses “walk” and “interact while walking” they avoid spaces with better global access and visibility; and that everyone in ICUs “walk” more in spaces with higher control over neighboring spaces. It is argued that such consistent behavioral patterns occur due to the structural similarities of spatial networks over and above the more general functional similarities of ICUs.

## 1. Introduction

In recent years, several studies on psychology, behavior and health use rigorous techniques and measures of “space syntax” describing the network properties of spaces in terms of visibility and accessibility within hospitals and hospital nursing units of different geometry, size, and functions. As a result, the findings of these studies in dealing with the relationships between the network of spaces, on the one hand, and psychological responses and behavior, on the other, are less context-dependent and more generalizable [1,2,3,4,5,6,7,8,9,10,11,12]. Several space syntax studies to date, including the ones reported here, show strong relationships between spatial network measures and environmental behavior and psychology. Hillier and Hanson [13] and Hillier [14] are the two early publications that discuss the theoretical reasons why the relationships between network measures and behaviors are found.

In general, the space syntax studies of nursing units use two generic measures of network centrality-integration and control [13] characterizing all the spaces in a unit, and one more specific measure of network centrality-targeted visibility [8] characterizing only a few selected spaces/objects of interests based on the objective patterns of visibility and accessibility in the unit. Despite differences, however, all these space syntax measures are developed based on the same mathematical foundations described in [13].

Simply defined, integration is a global network measure of centrality describing how a spatial element, which can be either 1- or 2-dimensional, is connected to all the other such elements in a layout. Spatial elements with higher integration are more easily visible and/or accessible from all the other similar elements in the layout; hence, they occupy more central positions in the system in terms of either visibility, accessibility, or both.

While integration is a global network measure, control is a local network measure of centrality that describes the degree to which a spatial element controls visual and/or physical access to its immediate neighboring spatial elements taking into account the number of alternative connections of each neighboring element. Hence, spatial elements with high control occupy more central positions within neighboring spaces in terms of how movement occurs among these neighboring elements.

In contrast to integration and control, the targeted visibility of a target, either a spatial element or an object, is computed based on the number of targets visible from a given target in relation to all the other similar targets in any given layout [8]. (See the next section for additional information about these space syntax techniques and measures).

In their earlier study using integration and control, Alalouch and Aspinall [1] investigated locational effects on perceived privacy in six different types of multi-bed units in the UK. A group of 79 subjects were asked to complete a questionnaire on privacy and to select preferred and disliked locations on plans of hospital wards. In the study, participants’ chosen locations for privacy showed a systematic relationship with spatial network properties of the unit layouts. Their choices for both high and low privacy locations were best represented by integration and control. At a unit level participants’ preference for greater privacy was for units with low integration and high control values; and within any unit, at a bed location level participants’ preference for privacy was in lower integration and lower control locations.

Later, using the additional data collected during the study reported in Alalouch and Aspinall [1], Alalouch *et al.* [2] investigated the effect of age, gender, previous experience of space and cultural background on people’s chosen spatial location for privacy. Spatial data for this more recent study were provided by Visibility Graph Analysis (VGA) of space syntax [15]. Findings indicated a universal preference for spatial location of privacy across culture, age and gender, and a specific significant difference for spatial location of privacy as a result of previous spatial experience. However, more studies are needed on privacy and intervisibility involving people with direct hospital unit experience in different cultures before the findings of the study can be generalized.

In another study, using space syntax techniques, Hendrich *et al.* [5] looked at the correlations between the integration values of individual spaces of the units and the dataset of the radio-frequency identification (RFID) movement patterns of 53 nurses, covering 143 nursing shifts, from 5 medical-surgical units. The results of their study showed that as integration of patient rooms increased, so did the frequency of shorter visits to these rooms and the total amount of time spent in these rooms. Later, using the data of the study reported by Hendrich *et al.* [5], Choudhary *et al.* [4] developed Generalized Linear Modeling (GLM) techniques to interpret the relationships between integration of spaces and nurses’ movement. In their study, Cai and Zimring [3] found strong correlations between the integration values of spaces and the frequency of nurses’ interaction and peer awareness in these spaces in two different wings of a neurological intensive care unit (ICU).

In a different kind of study, Trzpuc and Martin [10] used data from semi-structured interviews with end users and the more generic measures of centrality of space syntax to test several hypotheses on the potential functional benefits of visibility and accessibility in three medical-surgical units. They found that nurses’ perception of the potential functional benefits of visibility and accessibility did not match the potential benefits of these units identified using their network centrality values. While the finding is interesting, several confounding factors existed in the study. Therefore, the authors pointed out the need for additional studies before any definite conclusion could be drawn.

In addition, using the more generic measures of centrality of space syntax, Sagha Zadeh *et al.* [11] described the spatial relationships among clinical spaces in five medical-surgical units. The authors indicated that they measured movement distributions and identified possible conflicts with focus-demanding tasks, such as noise and interruptions in these units. However, they did not discuss how the data on movement distributions and possible conflicts were identified. Nevertheless, the authors identified several important areas in these units based on the study. Interestingly enough, some of these important areas such as report rooms, nourishment areas, and physician workspaces were not even included in the available healthcare design guidelines. The authors also identified that most caregivers move in patient corridors and nurses’ stations, posing the greatest possibility of interruptions by persons. They summarized the information in a visual diagram providing the “syntactic anatomy” of the units, and noted that the anatomy at this stage did not include many important design features including visibility, walking distance, scale, and number of beds and supply areas. They also noted that shadowing and observing of nurses would be necessary to customize the anatomy for individual cases in any future study.

Finally, using the more generic measures of centrality of space syntax, Kim and Lee [6] investigated how the techniques of space syntax could be applied to assess the Whole-life Target Value Design (TVD) of healthcare facilities. While TVD can be defined as a management practice that strives to eliminate waste by setting a target cost and by spurring innovation within project constraints [16], the Whole-life Target Value Design is a broad application of TVD toward facility operation and management, and user costs beyond initial costs of designing and building a facility. The authors used three hypothetical nursing units-shallow-plan, deep-plan and courtyard-plan-to determine which alternative is the most cost-efficient in terms of users. However, they do not define user costs in terms of financial value due to a lack of empirical evidence. Instead, they discuss user costs using such qualitative terms as high, medium and low. Based on the study, the authors found that the deep-plan has the lowest and the shallow-plan the highest user costs.

In contrast to the aforementioned studies using more general concepts of spatial centrality, Lu *et al.* [8] used the concept of targeted visibility in their study. The targeted visibility of such objects as patient beds in nursing units is important from a clinical perspective, because patient safety and medical outcomes often depend on how visible these beds are within a nursing unit [7]. In their study on a Neurological ICU, Lu *et al.* [8] found that the distribution of staff density was more strongly correlated with the centrality measures computed based on targeted visibility than it was with the generic centrality measures. They also found that nurses, especially when interacting, prefer positions that provide high visual access to multiple patients; and that doctors prefer positions that maximize their awareness of the surrounding environment. Based on the study, Lu *et al.* concluded that clinical staff members are tuned to different aspects of the visual structure of spaces based on their roles in hospital units.

In a later study, Lu and Zimring [9] used the concept of targeted visibility to define the targeted visibility index (TVI) in order to compare different nursing units. TVI is expressed as the ratio of the average of the targeted visibility values of all locations and the total number of targets in the units. TVI, thus, describes the degree to which an observer can see all the targets in the units. Lu and Zimring used this measure to characterize the three units used in the study reported by Trites *et al.* [17], and found that the circulation spaces in the radial unit had the highest TVI, followed by the circulation spaces of the double-corridor units and then by that of the single-corridor unit. Thus, forty years after Trites *et al.* [17], these authors were able to define what Trites *et al.* might have meant when they indicated that their radial unit had better visibility conditions than the double-corridor unit, which in turn had better visibility conditions than the single-corridor unit.

More recently, Lu *et al.* [7] reanalyzed Leaf *et al.* [18] data using TVI to describe the visibility of patient rooms in relation to ICU mortality. They found that among the sickest patients (those with Acute Physiology and Chronic Health Evaluation II > 30), visibility of patient rooms as measured by TVI accounted for 33.5% of the variance in ICU mortality (*p* = 0.049), thus providing additional support for the importance of the visibility of patients in relation to ICU outcomes.

In summary, the studies using space syntax techniques have been able to show why and how the network properties of spaces are important for behaviors such as movement and interaction, psychological responses such as privacy, and medical and economic outcomes in nursing units. Together, they provide a way to develop theories linking behavior, psychological responses, and spatial network that bear some resemblance to Gibson’s concept of *affordance* [19,20]. Gibson had offered this concept as an essential aspect of his theory of ecological perception, and argued that it is through the perception of affordances that animals perceive their environment, and that in any interaction between an animal and its environment, affordances allow the animal to perform certain actions with the environment. In general, positive affordances encourage human interactions, and negative affordances discourage them.

Later, Norman used the term “perceived affordability” describing the degree to which the affordance of an object maps onto its purpose [21,22]. If a chair does not allow the perceived affordance of “sit-ability”, then the natural mapping between its purpose and affordance does not exist. Since this absence of natural mapping breeds problems, one of the many goals of design research has been to understand how “perceived affordability” works in relation to design and its end users [23,24,25,26,27].

In reality, however, the affordance of an object appears to be more easily defined than that of an environment. Space syntax theories and techniques are useful in this regard, because they use “perceptual primitives” (*i.e.*, objectively defined spatial elements which are perceptually meaningful) to define the objective structures of the environment, and then study how much of the variance in observed behavior and psychological responses can be explained by these structures [28,29,30].

Yet, for the purposes of a theory explaining the effects of the network properties of spaces on behavior, psychological responses, and health in nursing units the number of space syntax studies reported in the literature remains insufficient. With the exception of Hendrich *et al.* [5] the reported studies are quite narrow in scope. Either they use only one or two case studies; or they use a very narrow set of predictor and dependent variables. In their study, using space syntax techniques, Hendrich *et al.* used RFID movement data from as many as 5 medical-surgical units; but their datasets did not contain enough behavioral information. As a result, their findings were limited only to the frequency and the length of visits by nurses in patient rooms indicating a need for more studies on behaviors in nursing units.

Therefore, to further investigate the importance of the network properties of spaces in relation to behaviors in nursing units, the aim of this correlational research was to study the relationships between three network measures and four interaction-related behaviors in ICUs with different specialty types, size, and layout. The research focused on interaction-related behaviors because they promote effective communication and collaboration; and previous studies have indicated that effective communication and collaboration are key factors in patient and staff outcomes in hospitals and ICUs [31]. In this regard, the research asked the following questions:

- Are the ICUs included in the study similar or different in terms of spatial network properties describing visibility and accessibility? Our hypothesis concerning this is that the properties of spatial network in these ICUs would be similar because all ICUs require better environmental visibility and accessibility for efficient and effective patient care.
- Are the observed patterns of interaction-related behaviors similar or different in the ICUs included in the study? Our hypothesis concerning this is that the observed behavioral patterns in the ICUs would be different from each other because of the differences in the clinical services they provide.
- Are the interaction-related behaviors of ICU users (excluding patients) correlated with spatial network properties? Do the correlations vary based on the category of the participants engaged in these behaviors? Our hypothesis concerning this is that some interaction-related behaviors in these units would show consistent relations to spatial network measures describing accessibility and visibility. This might occur because all users generally would take advantages of spatial visibility and accessibility in ICUs to provide patient care.

## 2. Method

### 2.1. Study Sample

**Figure 1:**
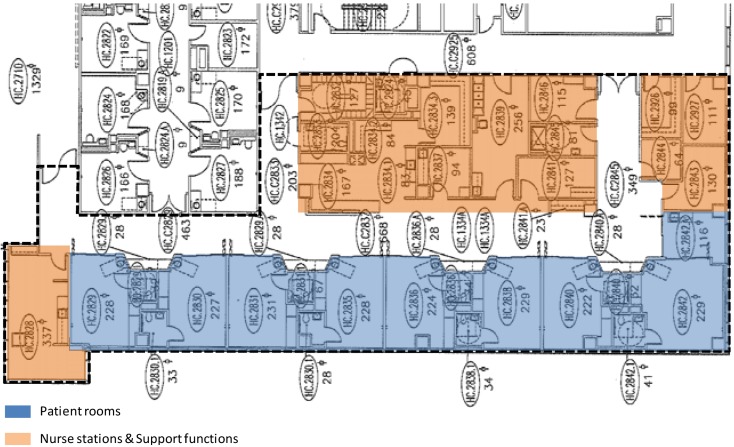
The 8-bed ICU-B.

**Figure 2:**
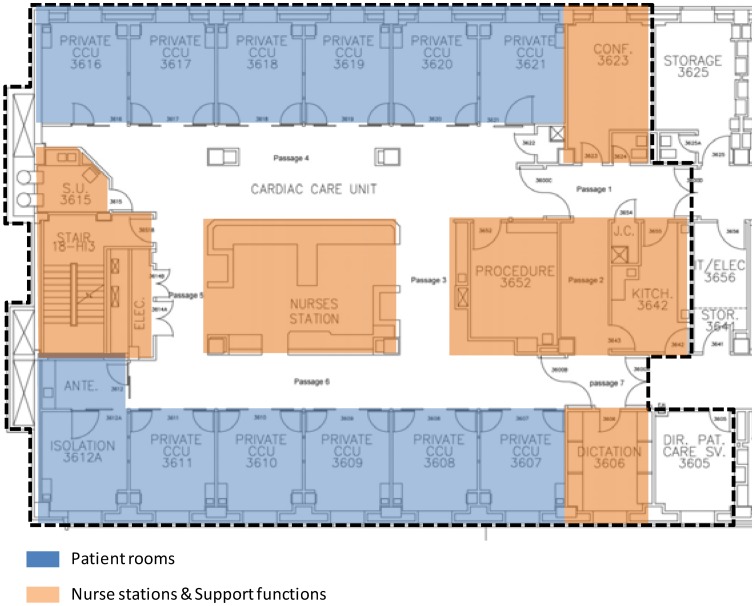
The 12-bed ICU-A.

**Figure 3:**
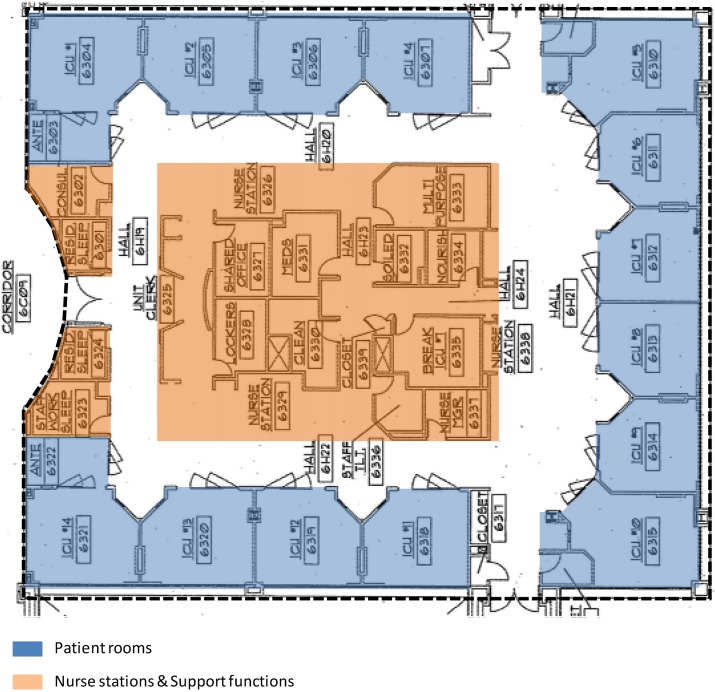
The 14-bed ICU-C.

**Figure 4:**
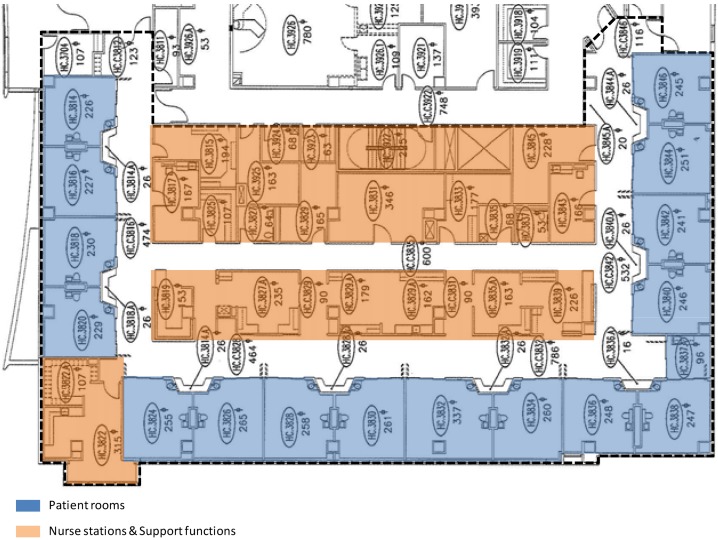
The 16-bed ICU-D.

The research was conducted at four adult ICUs (Figure 1, Figure 2, Figure 3 and Figure 4) in two major hospitals in a large metropolitan area. Two of these units are Coronary Care Units (ICU-A) and Cardiac Intensive Care Units (ICU-B), one is a Transplant Intensive Care Unit (ICU-C), and the fourth one is a Cardiothoracic Surgical Intensive Care Unit (ICU-D). ICU-B is an 8-bed unit; ICU-A a 12-bed unit; ICU-C a 14-bed unit; and ICU-D is a 16-bed unit.

### 2.2. Data Collection

After receiving approvals from the Institutional Review Boards of all involved institutions, data collection was conducted in two phases, which are discussed next.

#### 2.2.1. Spatial Analysis

In this phase, network properties of spaces in the four ICUs were described in terms of environmental visibility and accessibility using space syntax techniques. Over the years, space syntax has provided several techniques describing network properties of 1- or 2-D spatial elements in a layout. Included among them are the more traditional techniques of axial and convex map analysis [13,14], and the more recent techniques of visibility graph analysis (VGA) and angular segment analysis (ASA) [15,32]. While the traditional space syntax techniques simplify a layout to a network of few linear or convex elements, VGA avoids the simplification using connectivity patterns of visual fields available from all possible locations in a layout. ASA, in contrast, allows network analysis to take into account angular distances between nodes that the traditional axial map analysis does not allow. Therefore, both VGA and ASA are more robust than axial and convex map in relation to the finer aspects of spatial layouts.

For several technical reasons, however, this study uses only two kinds of network measures, derived based on the axial map analysis and the node network analysis (a modified version of the convex map analysis) techniques of space syntax. Among the reasons is that fact that VGA does not automatically characterize larger, discrete spatial or functional elements in a layout like the way an axial map analysis or a convex map analysis does. Therefore, it is not easy to use VGA to explain behaviors observed along a line or within a space. If many behaviors occur at many different locations, to identify the network properties of the locations of these observed behaviors using VGA can be rather daunting, if not impossible.

The study used the *Depthmap* software [33] for axial and node network analyses. In the axial map analysis, a floor layout is described as an axial map composed of the minimum number of axial lines needed to cover every space and complete every circulation ring in the layout. *Depthmap* computes the network measures of the axial lines and the axial map based on how these lines are connected with one another. In contrast, a floor layout is decomposed into a set of functionally distinct spaces in the node network analysis. *Depthmap* computes the network measures of the spaces and the layout based on how these spaces (or nodes) are connected with one another. Note here that in the traditional convex map analysis, a floor layout is decomposed into a set of convex spaces as opposed to a set of functionally distinct spaces for network analysis.

**Figure 5:**
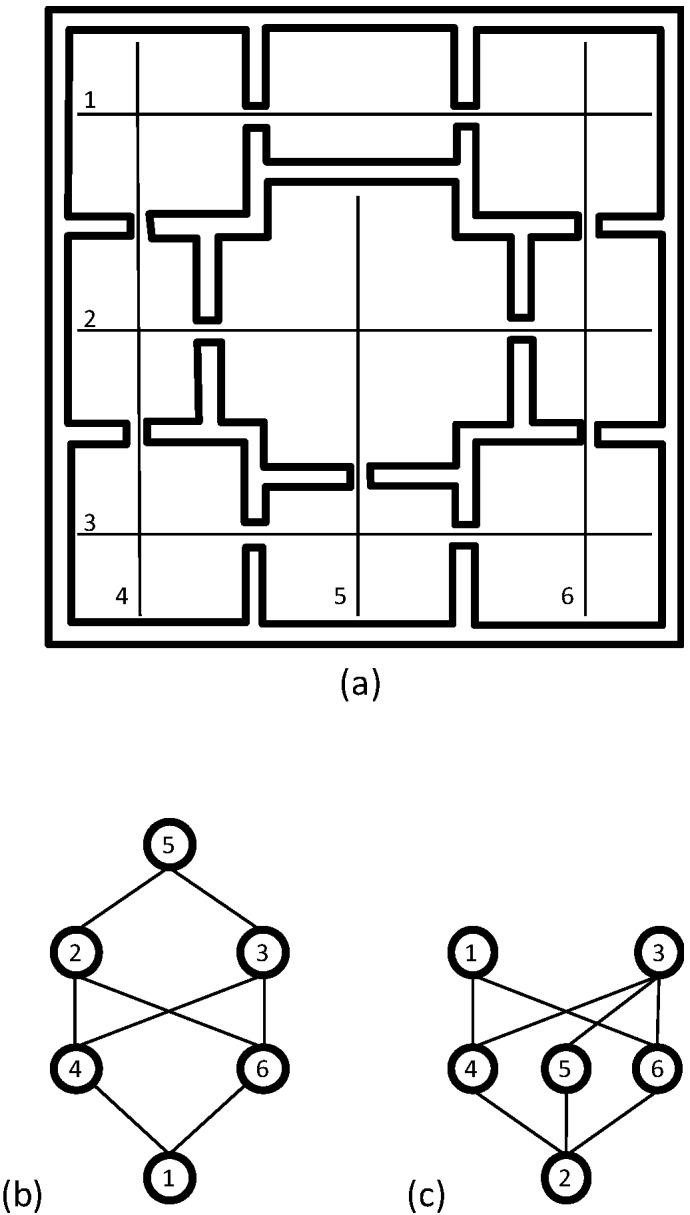
The basic concept of the axial map analysis: (**a**) A floor layout and its axial map; (**b**) The justified graph of axial line-1 shows that in order to get to all other axial lines from this line at least three steps are needed; (**c**) The justified graph of axial line-2 shows that in order to get to all other axial lines from this line only two steps are needed.

**Figure 6:**
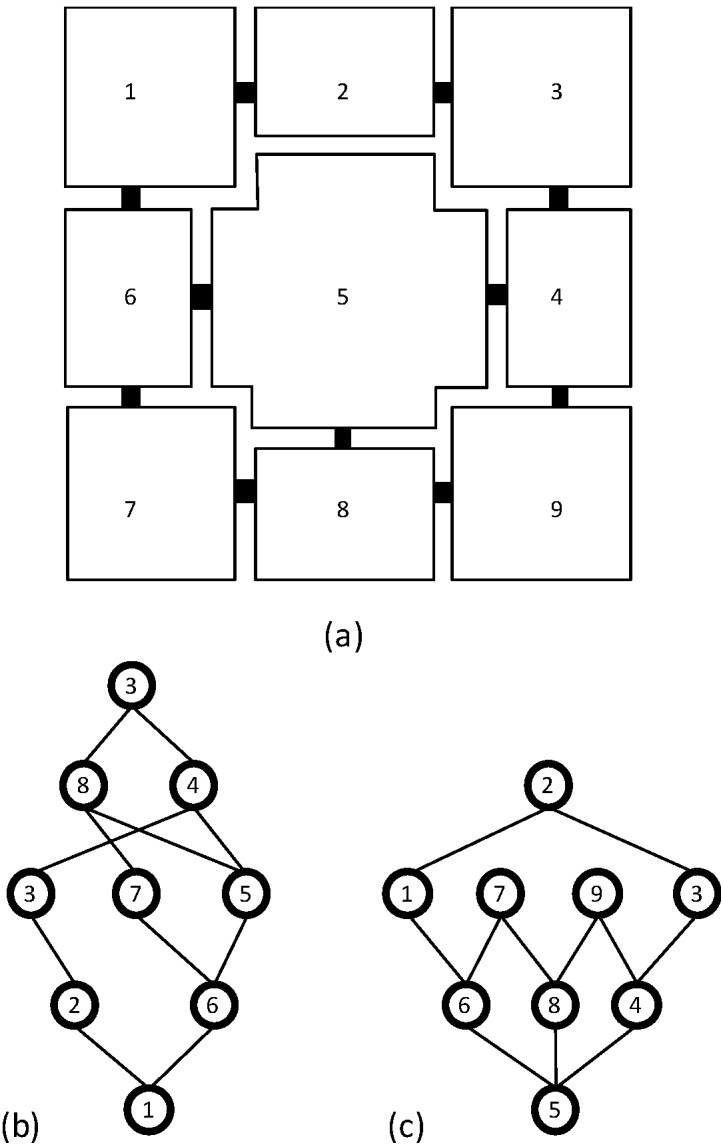
The basic concept of the node network analysis: (**a**) A floor layout and its functional spaces with their connections; (**b**) The justified graph of space-1 shows that in order to get to all other spaces from this space at least four steps are needed; (**c**) The justified graph of space-5 shows that in order to get to all other spaces from this space only three steps are needed.

How Space Syntax computes the network measures can easily be illustrated visually by graphs (Figure 5 and Figure 6). In the graph, the axial lines or spaces are treated as vertices or nodes and their intersections or connections as edges. In order to represent how a line or a space is connected to all the other lines or spaces in the layout, this graph can be rearranged using a line or space as the root vertex and all the other lines or spaces as vertices on successive layers defined based on the minimum number of lines or spaces one must use to reach them from the given line or space. The rearranged graph, also known as the justified-graph, will either look shallow with few layers if the line or space is well-connected to other lines or spaces, or it will look deep with more layers if the line or space is poorly connected to other lines or spaces. Space syntax computes most network measures based on the justified graphs drawn from every axial line or space.

Among many network measures of space syntax, this study used integration and control only. The integration value of a node in a graph is an algebraic function of the mean depth (MD) value of the node, which is the sum of the shortest distances between the node and all other nodes in the graph divided by the number of nodes in the graph less 1. In contrast, if a node in a graph has *n* immediate neighbors, then the node gives 1/*n* of its control to each of its immediate neighboring nodes. These values are then summed for each receiving node to give the control value of that node [13].

In this study, we expect to find more dynamic behaviors such as “walking” and “walking and interacting” (see below for definitions) in more integrated spaces or on more integrated lines in the ICUs. Since in a relatively small open environmental setting, such as the ones studied here, axial lines often undermine any sense of environmental control by crossing thresholds of several functionally distinct spaces, in this study we used node control rather than axial control. We expect to find more static behaviors such as “standing and interacting” and “sitting and interacting” (see next section for definitions) in spaces with more node control.

#### 2.2.2. Behavioral Observation

In this phase, interaction-related behaviors were observed in different spaces of the four ICUs to discover the patterns of spatial distribution of the behaviors in different spaces of these units. It was expected that these patterns would be different for each unit because each served a different specialty. Since the census in each of these ICUs was generally low during the weekends, the behavioral observations were conducted during the weekdays only. In general, eight to ten rounds of observations, each lasting about 20 to 30 min, were completed during a day shift with a thirty-minute break in-between any two consecutive observation rounds. The break was needed to avoid observing the same behavior more than one time, and to allow new spatial patterns of behaviors to develop in the unit.

The observations were made from one or more locations that had the most areas of a unit visible with the least impact on unit activities. During an observation round, the field observer observed a predefined set of behaviors in all visible spaces of an ICU among three categories of the population: (1) nurses (included nurses of all positions or ranks); (2) physicians (included physicians of all positions and ranks); and (3) others (included visitors, support staff, and everyone else other than nurses and physicians present on the floor at the time of observation).

The observer recorded four categories of interaction related behaviors, which included: (1) “walking” defined as individuals taking trips between spaces, thereby creating opportunities for chance encounters and interactions; (2) “walking and interacting” defined as individuals actively engaged with other individual/s for any amount of time during a trip from one space to another; (3) “standing and interacting” defined as individuals actively engaged with other individual/s for any amount of time standing next to others; and (4) “sitting and interacting” defined as individuals actively engaged with other individual/s for any amount of time while seated. The term “actively engaged” was used to refer to any activities that required at least two individuals to be involved in an activity. Examples of these activities may include talking to each other, looking at an image or a chart together with or without talking, attending a patient together with or without talking, *etc*.

Since individuals were the unit of observation in this study, every interaction was considered to be an event participated in by individuals who performed any one or some combinations of the aforementioned behaviors. In other words, individuals representing different population categories could be engaged in different interaction-related behaviors in any one discrete interaction event. For example, in a rare interaction event, a seated nurse could be talking to a standing physician while another physician responded to a query as she walked by. The field observer was trained to record behaviors performed by each individual separately during an interaction event. She recorded the frequency of occurrences of these behaviors, the category and number of people involved in these occurrences, and the location of these occurrences on a floor layout drawing of each unit during observation rounds using paper and pencil.

40 (±5) rounds of behavioral observations were completed over a week in each ICU. Altogether, 160 rounds of observation were completed at the four sites. The observations included 148 spaces in the four ICUs. 53 of these spaces were patient rooms, 19 were spaces within nurse stations, 26 were circulation spaces, and the rest were spaces serving different support functions.

### 2.3. Data Analysis

Data analysis was conducted in two phases using IBM SPSS Statistics 20 [34], which are discussed next.

#### 2.3.1. Analysis of Rank Order of Different Categories of Spaces in the Units

In this phase of the data analysis, four important categories of spaces—patient rooms, nurse stations, circulation spaces, and support functions—were ranked in all four units using axial integration, node integration, and node control (see next section). The node integration and control values are assigned to individual spaces; therefore, the means of these values of all the spaces of a category were used for rank orders. In contrast, the axial integration values are assigned to axial lines that often cut across multiple spaces. Therefore, first, the mean of the axial integration values of all the axial lines crossing a space was assigned as the integration value of the space. Following this, the mean of all the assigned integration values of all the spaces of a category was used for rank orders.

It was noted earlier that the two integration values describe the global patterns of spatial relations, and the node control values describe the local patterns of spatial relations in the layout. Therefore, the rank orders of spaces based on these measures described similarities and differences of these units at the local and global levels of spatial networks. These rank orders were then used for explaining similarities or differences in the patterns of relationships between network measures and interaction-related behaviors in the units.

#### 2.3.2. Correlational Analysis

In this phase of data analysis, the relationships between network measures and interaction-related behaviors in the ICUs were studied using correlational analysis to find out if these relationships were consistent despite unit differences. First, the correlational analysis considered all observed spaces in the ICUs to find out the relationships between network measures and interaction-related behaviors at the unit level based on population category (Table 1). Following this, the correlational analysis considered observed spaces with similar functions to find out the relationships between network measures and interaction-related behaviors for the spaces of each functional category, again, based on population category (Table 1). Only three of the four functional categories of spaces–patient rooms, nurse stations, and circulation spaces–were considered in this phase of analysis. Spaces providing support functions (e.g., soiled room, clean linen, nourishment, med-supply, offices, conference rooms, and kitchen) were not included in our analysis because these spaces were not consistently available for observation.

Once the axial integration, node integration and node control values of the spaces in each ICU were determined following the methods described in the previous section, first, the correlational analysis included these network values of all the observed spaces and the interaction-related behaviors in these spaces. Following this, the correlational analysis included the network values of the observed spaces of a category and the behaviors observed in these spaces.

**Table 1:** Correlations between Interaction-Related Behaviors and Network Measures in ICUs.

		Unit level (*n* = 148)			Patient rooms (*n* = 53)			Nurse stations (*n* = 19)			Circulation spaces (*n* = 26)		
	Observed Behaviors	Axial Integration	Node Integration	Node Control	Axial Integration	Node Integration	Node Control	Axial Integration	Node Integration	Node Control	Axial Integration	Node Integration	Node Control
Nurses	Walking		-0.205*	0.385**					0.743**		0.541**		
	Standing and Interacting								0.564*				
	Walking and Interacting	−0.165*						−0.633**				−0.442*	
	Sitting and Interacting	0.194*	0.301**					0.575*			0.605**		
	Total Observed					−0.335*	−0.345*		0.471*				
Physicians	Walking		−0.202*	0.351**									
	Standing and Interacting						−0.276*		0.505*				
	Walking and Interacting		−0.189*										
	Sitting and Interacting	0.387**						0.538*			0.781**		
	Total Observed	0.290**					−0.321*				0.485*		
Others	Walking		−0.203*	0.480**				−0.657**	0.606**				
	Standing and Interacting												0.472*
	Walking and Interacting		−0.165*			−0.340*		−0.494*				−0.465*	
	Sitting and Interacting			−0.204*			−0.379*		−0.631**		0.643**		
	Total Observed					−0.394**	−0.272*						

** Correlation is significant at the 0.01 level (2-tailed); * Correlation is significant at the 0.05 level (2-tailed).

## 3. Results

### 3.1. Results Based on the Rank Order of Different Categories of Spaces in the Units

First, the rank orders of mean axial integration of different categories of spaces in the four units were studied. Recall that the axial integration value of a space describes how visible and accessible the space is from all the other spaces in a spatial layout when the layout is considered as an axial map (*i.e.*, a network of lines of movement and sight). Therefore, a rank order of different categories of spaces using this value may help compare how easily accessible and visible the spaces of any given categories are in the networks of the lines of movement and sight of the layout. According to the rank orders shown below, ICU-B and ICU-C are similar; ICU-A is almost similar to ICU-B and ICU-C; and ICU-D is quite different from the other three:

ICU-A: (1) Circulation → (2) Patient Rooms → (3) Nurse Stations → (4) Support functions
ICU-B: (1) Circulation → (2) Nurse Stations → (3) Patient Rooms → (4) Support functions
ICU-C: (1) Circulation → (2) Nurse Stations → (3) Patient Rooms → (4) Support functions
ICU-D: (1) Circulation → (2) Patient Rooms → (3) Support functions → (4) Nurse Stations

Second, the rank orders of mean node integration of different categories of spaces in the four units were studied. Again, recall that the node integration value of a space describes how accessible the space is from all the other spaces in a spatial layout when the layout is considered as a network of discretely defined spaces. Therefore, a rank order of different categories of spaces using this value may help compare how easily accessible the spaces in any given categories are in the network of spaces of the layout. According to the rank orders shown below, these units are similar:

ICU-A: (1) Circulation → (2) Nurse Stations → (3) Patient Rooms → (4) Support functions
ICU-B: (1) Circulation → (2) Nurse Stations → (3) Patient Rooms → (4) Support functions
ICU-C: (1) Circulation → (2) Nurse Stations → (3) Patient Rooms → (4) Support functions
ICU-D: (1) Circulation → (2) Nurse Stations → (3) Patient Rooms → (4) Support functions

Finally, the rank orders of mean node control of different categories of spaces in the four units were studied. Control, as defined in a previous section, describes the degree to which a space controls visual and/or physical access to its immediate neighboring spaces taking into account the number of alternative connections of each neighboring space. Hence, spaces with high control occupy more central positions within neighboring spaces in terms of how movement occurs among these neighboring spaces. According to the rank orders shown below, three out of the four units-ICU-A, ICU-B, and ICU-C-are almost similar:

ICU-A: (1) Circulation → (2) Nurse Stations → (3) Patient Rooms → (4) Support functions
ICU-B: (1) Circulation → (2) Nurse Stations → (3) Support functions → (4) Patient Rooms
ICU-C: (1) Circulation → (2) Nurse Stations → (3) Patient Rooms → (4) Support functions
ICU-D: (1) Circulation → (2) Support functions → (3) Nurse Stations → (4) Patient Rooms

In summary, the analysis of the floor plans of the four units showed no differences among the four units based on node integration. At least three out of the four units-ICU-A, ICU-B, and ICU-C-were noticeably similar based on axial integration and node control.

The aforementioned findings are interesting in light of the fact that the layouts of the units are significantly different in terms of shape, size, and composition. The smallest 8-bed ICU-B (Figure 1) is a single-corridor layout with patient rooms on one side, and the nurse station and support spaces on the other side of the corridor. For every two rooms, there is one observation station in this unit. The 12-bed ICU-A (Figure 2) is a rectangular double-corridor racetrack type layout with patient rooms on either side of a core that includes the nurse station and other support areas. The 14-bed ICU-C (Figure 3) is a deep racetrack layout. Unlike ICU-A, it has a central core of nursing stations and support areas surrounded on three sides by patient rooms. Instead of one nurse station, the unit has one smaller and two larger substations placed around support spaces. The unit also has one observation station for every two patient rooms. The largest 16-bed ICU-D (Figure 4) is a rectangular racetrack type layout as well, with the nurse station at the center. However, unlike a regular racetrack type unit (e.g., ICU-A), this unit has patient rooms on the three sides and support areas on the fourth side of the nurse station. As a result, one of the main corridors of the unit serves the nurse station and support areas only; thus, making the functional distribution of the unit very different from the other units.

One wonders, how would the aforementioned observed similarities of the spatial network properties (or, simply structural similarities) of the four units affect interaction-related behavioral patterns in these units? Could these network similarities explain behavioral patterns in these units, regardless of their differences not only in terms of functions but also in terms of layout, shape, size, and compositions?

### 3.2. Results of Behavioral Observations

**Figure 7:**
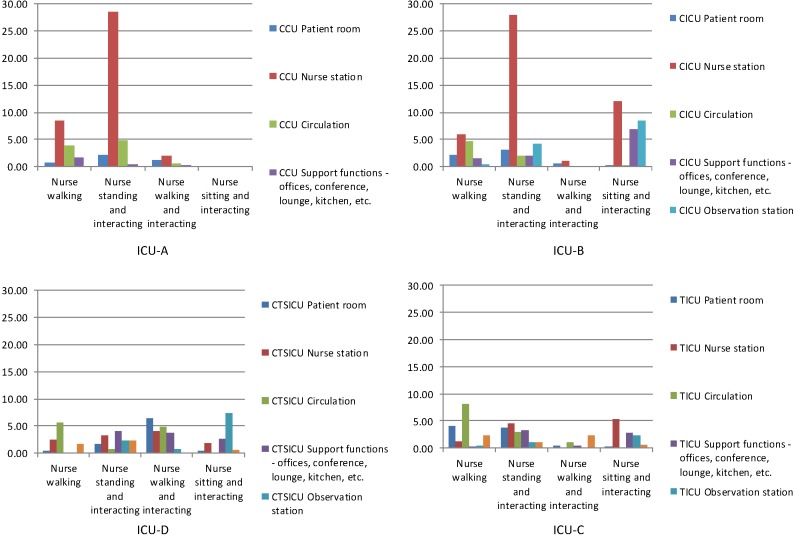
Total interaction-related behaviors per observation among nurses observed in different space in the four units.

**Figure 8:**
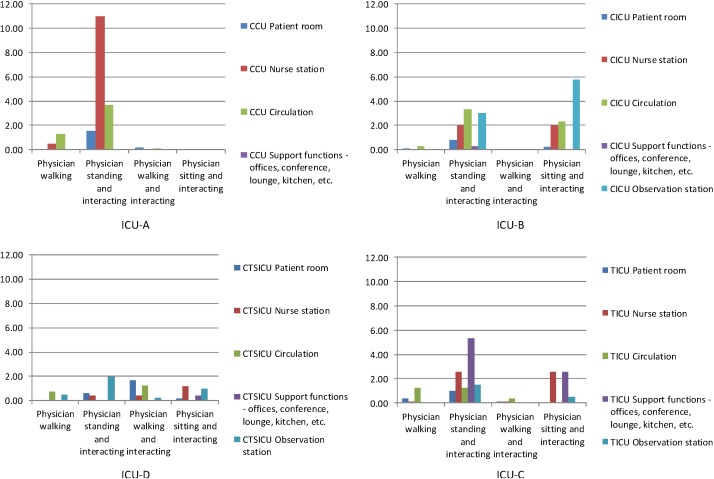
Total interaction-related behaviors per observation among physicians in different space in the four units.

**Figure 9:**
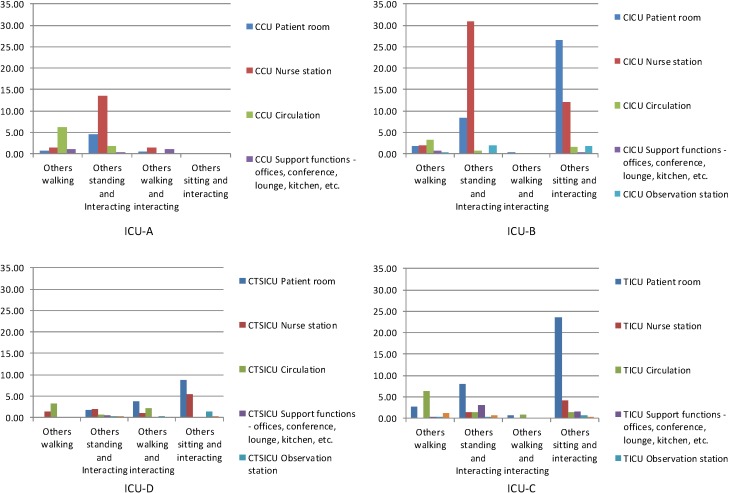
Total interaction-related behaviors per observation among others in different space in the four units.

As was noted in the data collection section above, 40 (±5) rounds of behavioral observations were completed over a week in each ICU. The histograms in Figure 7, Figure 8 and Figure 9 show the numbers of different types of interaction-related behaviors per observation among nurses, physicians, and others in different categories of spaces of each of the four units.

The histograms in Figure 7 show the distribution patterns of the interaction-related behaviors per observation among nurses in the four units. As expected, the overall distribution patterns of the behaviors among are different in different units. However, some similarities between ICU-A and ICU-B are observed. In ICU-A and ICU-B nurse stations were the most active spaces with regards to any interaction-related behaviors among nurses; and “standing and interacting” among nurses in the nurse stations was by far the most frequently observed interaction-related behavior in these units. In contrast, in both ICU-D and ICU-C nurses’ interaction-related behaviors occurred less frequently, and were distributed over several types of spaces.

The histograms in Figure 8 show the distribution patterns of interaction-related behaviors among physicians in the four units. Again, the overall distribution patterns of the behaviors among physicians are different in different units. In ICU-D interaction-related behaviors occurred least. In ICU-C interaction-related behaviors were more frequent than in ICU-D. In ICU-B only two of the four interaction-related behaviors were observed. In each of these three ICUs interaction-related behaviors were distributed over several spaces. In ICU-A interaction-related behaviors were limited to fewer spaces, and “standing and interacting” was by far the most frequently observed behavior. In ICU-A and ICU-B physicians were most frequently engaged in “standing and interacting” behaviors. However, in ICU-A “standing and interacting” occurred most frequently in the nurse station; whereas in ICU-B the behavior was distributed among different types of spaces. In ICU-B observation stations were also used frequently by physicians.

The histograms in Figure 9 show the distribution patterns of interaction-related behaviors among others (*i.e.*, people who were not nurses and physicians) in the four units. Once again, the overall distribution patterns of the behaviors among others are different in different units. In ICU-A and ICU-B “standing and interacting” was the most frequently observed behaviors among others; and nurse stations were the most active spaces in terms of this behavior. In the patient rooms of ICU-B, ICU-D and ICU-C, “Sitting and interacting” was also observed quite frequently. In fact, in ICU-C this was the most observed behavior.

### 3.3. Results of Correlational Analyses

#### 3.3.1. Results of Correlational Analyses between Axial Measures and Behaviors: Unit Level

Axial integration and nurses’ behaviors: Axial integration showed a significant but weak positive correlation with “sitting and interacting” among nurses (*r* (148) = 0.194, *p* = 0.032), indicating that nurses might have interacted more when seated along more integrated axial lines. In contrast, axial integration showed a significant but weak negative correlation with “walking and interacting” among nurses (*r* (148) = −0.165, *p* = 0.046), indicating that nurses might have interacted less when walking along more integrated axial lines.

Axial integration and physicians’ behaviors: Axial integration also showed a significant positive correlation with “sitting and interacting” among physicians (*r* (148) = 0.387, *p* = 0.000), indicating that physicians interacted more when seated along more integrated axial lines. The fact that the correlation was stronger for physicians than it was for nurses suggests that axial integration affected “sitting and interacting” among physicians more than it affected the same behavior among nurses. In addition, the total number of physicians observed in any space was also significantly correlated with axial integration (*r* (148) = 0.29, *p* = 0.000), indicating that more interaction-related behaviors among physicians occurred along more integrated axial lines.

Axial integration and others’ behaviors: Axial integration showed no significant correlation with any one of the interaction-related behaviors among others, indicating that axial integration had no relationship with interaction-related behaviors among others.

#### 3.3.2. Results of Correlational Analyses between Node Measures and Behaviors: Unit Level

Node measures and nurses’ behaviors: “Walking” among nurses was negatively correlated with node integration (*r* (148) = −0.205, *p* = 0.012), but was positively correlated with node control (*r* (148) = 0.385, *p* = 0.000). These findings indicate that nurses generally walked less in spaces with higher node integration, but walked more in spaces with higher node control. Node integration was also positively correlated with “sitting and interacting” among nurses (*r* (148) = 0.301, *p* = 0.001), indicating that nurses interacted more while seated in spaces with higher node integration.

Node measures and physicians’ behaviors: Like nurses, “walking” among physicians was negatively correlated with node integration (*r* (148) = −0.202, *p* = 0.014), but was positively correlated with node control (*r* (148) = 0.385, *p* = 0.000). These findings indicate that, like nurses, physicians generally walked less in spaces with higher node integration, but walked more in spaces with higher node control. Node integration also showed a significant negative correlation with “walking and interacting” among physicians (*r* (148) = −0.189, *p* = 0.022), indicating physicians interacted less as they walked in spaces with higher node integration.

Node measures and others’ behaviors: Like nurses and physicians, “walking” among others was also negatively correlated with node integration (*r* (148) = −0.203, *p* = 0.013), but was positively correlated with node control (*r* (148) = 0.48, *p* = 0.000). These findings indicate that others generally walked less in spaces with higher node integration, but walked more in spaces with higher node control.

While node integration showed a significant negative correlation with “walking and interacting” (*r* (148) = −0.165, *p* = 0.045), node control showed a significant negative correlation with “sitting and interacting” (*r* (148) = −0.204, *p* = 0.024) among others. These findings indicate that while walking others interacted less in spaces with higher node integration, and while seated others interacted less in spaces with higher node control.

#### 3.3.3. Results of Correlational Analysis between Axial and Node Measures and Behaviors: Spatial Category Level

Patient rooms: Axial integration showed no significant correlation with any one of the interaction-related behaviors among nurses, physicians and others in patient rooms, indicating that interaction-related behaviors in patient rooms were not affected by axial integration of these rooms.

In contrast, node integration showed significant negative correlations with all observed behaviors among nurses (*r* (53) = −0.335, *p* = 0.014), “walking and interacting” among others (*r* (53) = −0.340, *p* = 0.013), and with all observed behaviors among others (*r* (53) = −0.394, *p* = 0.004); and node control showed significant negative correlations with all observed behaviors among nurses (*r* (53) = −0.345, *p* = 0.011), “standing and interacting” among physicians (*r* (53) = −0.276, *p* = 0.045), all observed behaviors among physicians (*r* (53) = −0.321, *p* = 0.019), “sitting and interacting” among others (*r* (53) = −0.379, *p* = 0.014), and with all observed behaviors among others (*r* (53) = −0.272, *p* = 0.049).

Nurse stations: Axial integration showed a significant and moderately strong negative correlation with “walking and interacting” (*r* (19) = −0.633, *p* = 0.004), and a significant and moderately strong positive correlation with “sitting and interacting” (*r* (19) = 0.575, *p* = 0.016) among nurses in nurse stations. It showed a significant positive correlation with “sitting and interacting” (*r* (19) = 0.538, *p* = 0.026) among physicians in nurse stations. Finally, axial integration showed a significant and moderately strong negative correlation with “walking” (*r* (19) = −0.657, *p* = 0.002), and a significant negative correlation with “walking and interacting” (*r* (19) = −0.494, *p* = 0.032) among others in nurse stations.

Whereas node control did not show any correlations, node integration showed several significant correlations with interaction-related behaviors among nurses, physicians, and others in nurse stations of these units. Node integration showed a significant and strong positive correlation with “walking” (*r* (19) = 0.743, *p* = 0.000), a significant and moderately strong positive correlation with “standing and interacting” (*r* (19) = 0.564, *p* = 0.012), and a significant positive correlation with all observed behaviors (*r* (19) = 0.471, *p* = 0.042) among nurses in nurse stations. It also showed a significant positive correlation with “standing and interacting” (*r* (19) = 0.505, *p* = 0.027) among physicians in nurse stations. Finally, node integration showed a significant and moderately strong positive correlation with “walking” (*r* (19) = 0.606, *p* = 0.006), and a significant and moderately strong negative correlation with “sitting and interacting” (*r* (19) = −0.631, *p* = 0.007) among others in nurse stations.

Circulation spaces: Axial integration showed several significant positive correlations with behaviors in circulation spaces of the units. In particular, axial integration showed significant positive correlations with “walking” and “sitting and interacting” among nurses (*r* (26) = 0.541, *p* = 0.004, and *r* (26) = 0.605, *p* = 0.006, respectively); “sitting and interacting” among physicians and the total number of physicians observed (*r* (26) = 0.781, *p* = 0.000 and *r* (26) = 0.485, *p* = 0.012, respectively); and “sitting and interacting” among others (*r* (26) = 0.643, *p* = 0.003). These findings indicate that better environmental visibility and accessibility at the global level increased many interaction-related behaviors among all categories of people in the circulation spaces of the units.

Among node measures, node integration showed significant negative correlations with “walking and interacting” among nurses and others (*r* (26) = −0.442, *p* = 0.024 and *r* (26) = −0.465, *p* = 0.017, respectively); and node control showed a significant positive correlation with “standing and interacting” among others (*r* (26) = 0.472, *p* = 0.015) in circulation spaces. To be more precise, node integration had shown significant correlations in two instances out of 15; and node control had shown significant correlations in one instance out of 15 (see the last to columns of Table 1). Fewer numbers of correlations would, therefore, indicate that node integration and control had fewer relationships with interaction-related behaviors in circulation spaces of these units.

## 4. Discussion

### 4.1. Rank Order of Different Categories of Spaces in the Units

All ICUs require sufficient environmental visibility and accessibility for efficient and effective patient care. Therefore, it was hypothesized that the four ICUs included in this study would show similarities in spatial network properties describing environmental visibility and accessibility. The spatial layout analysis of the ICUs using the network measures of space syntax confirmed the hypothesis. Despite differences in specialty type, unit size, unit layout, and the distribution patterns of interaction-related behaviors, the ICUs included in the study showed underlying structural similarities defined by spatial networks.

The underlying structural similarities found here in the ICUs are also found in other designed environments of different size, shape and geometry [13,14,35]. The literature has termed these similarities as “spatial genotypes”, and has argued that they are fundamental in the way designed environments representing a “type” reproduce social and cultural knowledge [14,35]. In this sense, the findings of this study are not surprising. They only reinforce the fact that all ICUs must embody a shared body of commonly-held programmatic knowledge in their spatial structures regardless of specialty type, unit size, unit geometry, and behaviors to represent a particular “type” of environmental setting. Without such a shared body of spatial knowledge, designers may not be able to design ICUs and users may not be able to use them.

The importance of the spatial similarities among the four ICUs is further emphasized by the fact that many significant correlations between spatial network measures and interaction-related behaviors among nurses, physicians, and others were found in these units. It is quite possible that these correlations were an artifact of the spatial genotypes of these ICUs over and above the similar functional needs of different groups of users. If our interpretations of the environment-behavior relationships found in this study are correct, then it can be argued that “spatial genotypes” are an essential component of Gibson’s concept of affordance [20]. That is because they help define the inherent conditions or qualities of the environment (*i.e.*, affordances) that provide users with opportunities for taking certain actions in and with the environment.

### 4.2. Behavioral Observations

The four ICUs included in the study were different in terms specialty type, layout, shape, size, and composition. Therefore, it was hypothesized that the patterns of distribution of interaction-related behaviors in these units would be different. The findings of the observational study confirmed the hypothesis. In general, “standing and interacting” behaviors among different population groups—nurses, physicians, and others—were concentrated in one or two areas such as the nurse stations in ICU-A and ICU-B. In contrast, these behaviors were distributed over several areas in ICU-D and ICU-C. These differences in the distribution patterns of “standing and interacting” behaviors might have been due to the nature of clinical care given to patients in these units. In ICU-A and ICU-B, care regimens are relatively routinized and predictable. As result, “standing and interacting” occurred more predictably in some areas of ICU-A and ICU-B. In contrast, “standing and interacting” occurred more randomly in ICU-D and ICU-C patients in relation to more unpredictable and less routinized care regimens. However, it is also possible that ICU-D (16-bed) and ICU-C (14-bed) provided more opportunities for interaction than ICU-A (12-bed) and ICU-B (8-bed), simply because they were larger. This would then raise many interesting questions, such as: How much of the observed variations in behavioral patterns were due to the specialty type of these ICUs and how much due to their differences in size? Is it possible that at a certain threshold defined by unit size we would always expect a significant change in the distribution of interaction-related behaviors regardless of unit specialty? If so, where is this threshold? Is it always found between 12 and 14 as was observed in this study, or can this change in distribution also be found for other sizes? Is it possible that the differences in interaction-related behavior occur only due to the differences in specialty types, and that such differences have nothing to do with the size of a unit? Again, more studies on more ICUs of different specialty types and sizes are needed to answer these questions.

### 4.3. Correlational Analysis

The findings of the correlational analysis indicated many consistent relationships between network measures and interaction-related behaviors in these ICUs despite unit differences and differences in the distribution patterns of the behaviors.

Among other things, the findings showed that when nurses and physicians spent time “sitting and interacting” in these units they chose locations with higher axial integration (*i.e.*, axial lines with better global environmental visibility and accessibility), probably to help maintain a level of environmental awareness that was required in the intense environment of ICUs.

The findings also showed that correlations between “sitting and interacting” and axial integration were stronger in circulation spaces and nurse stations than they were at the unit level suggesting an interaction effect of these of spaces on the relationship. The interaction effect might have been due to any additional “sitting and interacting” behaviors generated by the observation stations that were located along circulation spaces, and by the technology and furniture that were available in the nurse stations of these ICUs.

In addition, the findings also showed that axial integration was negatively correlated with “walking and interacting” among nurses, and node integration was negatively correlated with “walking” among nurses. These findings contradicted our expectations that dynamic behaviors would be positively correlated with axial integration, and static behaviors would be positively correlated with node integration. Instead, they support a more general idea that “walking” or “walking and interacting” among nurses occurred less in more globally connected spaces regardless of whether the network was defined by functionally distinct spaces or by a set of axial lines.

Interesting, however, was the fact that “walking” among nurses, physicians and others was negatively correlated with node integration, but was positively correlated with node control. In other words, everyone in ICUs walked more in those spaces that provided better control over neighboring spaces, and less in those that provided better global visibility and accessibility. Though the findings were unexpected, the reason for this could have been that everyone in ICUs was generally more interested in taking opportunities that became available in neighboring spaces and therefore walked more in spaces with higher node control. Examples of such opportunities may include asking the physician or the nurse a question who is attending a patient next door, or seeking help of a nurse who is working on a computer in a nearby space. Taking advantages of such opportunities generally are easier from spaces with higher node control, simply because these spaces have better control over the neighboring spaces.

Finally, network measures had different relationships with interaction-related behaviors in different categories of spaces. For example, axial integration had no relationship with interaction-related behaviors in patient rooms; node control had no relationship with the behaviors in nurse stations; and node integration and control had very weak relationships with the behaviors in circulation spaces. Except for the fact that axial integration had positive correlations with “sitting and interacting” among nurses and physicians, network measures also had different correlations with interaction-related behaviors among different population categories. In simple words, the relationships of network measures with interaction-related behaviors depended on both spatial characteristics and population category.

## 5. Conclusions

This study looked at the effects of the network properties of spaces on the interaction-related behaviors among different user groups in four ICUs of different size, shape, layout, and specialty type. Systematic field observations showed significant differences in the spatial distribution of interaction-related behaviors in the ICUs. Yet, several consistent relationships between interaction-related behaviors and environmental accessibility and visibility defined using different local and global network measures were found. A remarkable degree of similarity in environmental visibility and accessibility among different categories of spaces in these units, as was shown in the rank order of the network measures of these spaces, could have been one of the reasons why interaction-related behaviors showed consistent relationships with network measures in these units. This is important in light of the fact that the need for better environmental visibility and accessibility for efficient and effective patient care may not diminish in ICUs in the near future, even though shifts in patient profiles, care delivery models, technology, labor force, *etc.* will continue to change how critical care is provided in ICUs.

It is also important to note here that despite any structural similarities and any consistent relationships between network measures and interaction-related behaviors in the ICUs, spatial distribution patterns of interaction-related behaviors of different user groups were different in different ICUs. In other words, different categories of users used spaces differently for their interactions in these units with somewhat similar structures of spatial networks. The findings, therefore, suggest that even though some commonly held programmatic knowledge is embedded in spatial networks in a similar way in different ICUs, such embedding is flexible enough to accommodate functional variations among different user groups. They also suggest that design interventions that require no changes in spatial network can be used to encourage or discourage interaction-related behaviors in ICU spaces.

To conclude, this study linking network properties of spaces to interaction-related behaviors in ICUs provides support for the importance network properties for behavior and psychological responses in nursing units. It also provides support for the concept of environmental affordance, for it shows many consistent relationships between objectively defined spatial network measures and interaction-related behaviors in ICUs despite unit differences and differences in the distribution patterns of the behaviors. Among them are the fact that spatial networks were used different user groups to maintain environmental awareness in ICUs; that certain network measures remained unchanged for certain categories of spaces in every ICU; and that the affordance of spaces with same network properties were often enhanced using additional furniture and fixtures to improve interaction-related behaviors.

Despite its contributions, the limitations of the study are clear. Even though the study included more interaction-related behaviors than any previous studies in nursing units using space syntax techniques, the data were limited to *who* participated in these interactions, *where* they participated, *how frequently* they participated, and *what* they did (moving, sitting, or standing) as they interacted. In future studies, it would be necessary to learn *when*, *why*, and *for how long* they participated in these interactions related behaviors. Observing when interactions occur should help us learn if some interaction-related behaviors are dependent on the time of the day. Knowing why interactions occur may help us learn their purpose and type. Some interactions are social in nature, while others are task-related. Some interactions are interruptions, while others are not. Knowing the durations of interactions may help us determine their spatial and technological needs. While shorter interactions can easily occur in corridors, longer interactions may require dedicated spaces.

With regard to space syntax techniques and measures, this study used only a few of many that are available. Each of these techniques and measures describes different aspects of spatial networks. Already in previous studies, visibility graph analysis (VGA) and targeted visibility analysis have shown usefulness for behavioral and psychological studies in nursing units. In future studies, it would be necessary to study the usefulness of these other techniques and measures for interaction-related behaviors in nursing units.

Finally, a few words need to be said on the bivariate correlation analysis used here to detect the relationship between a network variable and the frequency of an interaction-related behavior in ICUs. Though the analysis helped quantify the strength of the linear relationship between any two variables, in the future one should consider regression models to see if the frequency of an interaction-related behavior can be predicted by one or more spatial network variables. In addition, though the data were analyzed based on space and population categories to eliminate possible inflation in correlation due to subgroup effects, future studies should also consider gender, education, experience, and/or age to eliminate additional grouping effects within space and population categories.
